# Current Knowledge on *Porcine circovirus 3* (PCV-3): A Novel Virus With a Yet Unknown Impact on the Swine Industry

**DOI:** 10.3389/fvets.2018.00315

**Published:** 2018-12-12

**Authors:** Francini Klaumann, Florencia Correa-Fiz, Giovanni Franzo, Marina Sibila, José I. Núñez, Joaquim Segalés

**Affiliations:** ^1^CAPES Foundation, Ministry of Education of Brazil, Brasília, Brazil; ^2^IRTA, Centre de Recerca en Sanitat Animal (CReSA, IRTA-UAB), Campus de la Universitat Autònoma de Barcelona, Barcelona, Spain; ^3^Department of Animal Medicine, Production and Health (MAPS), University of Padua, Padua, Italy; ^4^UAB, Centre de Recerca en Sanitat Animal (CReSA, IRTA-UAB), Campus de la Universitat Autònoma de Barcelona, Barcelona, Spain; ^5^Departament de Sanitat i Anatomia Animals, Facultat de Veterinària, Universitat Autònoma de Barcelona, Barcelona, Spain

**Keywords:** *Porcine circovirus 3*, domestic pig, wild boar, infection, epidemiology

## Abstract

*Porcine circovirus 3* (PCV-3) is a recently described virus belonging to the family *Circoviridae*. It represents the third member of genus *Circovirus* able to infect swine, together with PCV-1, considered non-pathogenic, and PCV-2, one of the most economically relevant viruses for the swine worldwide industry. PCV-3 was originally found by metagenomics analyses in 2015 in tissues of pigs suffering from porcine dermatitis and nephropathy syndrome, reproductive failure, myocarditis and multisystemic inflammation. The lack of other common pathogens as potential infectious agents of these conditions prompted the suspicion that PCV-3 might etiologically be involved in disease occurrence. Subsequently, viral genome was detected in apparently healthy pigs, and retrospective studies indicated that PCV-3 was already present in pigs by early 1990s. In fact, current evidence suggests that PCV-3 is a rather widespread virus worldwide. Recently, the virus DNA has also been found in wild boar, expanding the scope of infection susceptibility among the *Suidae* family; also, the potential reservoir role of this species for the domestic pig has been proposed. Phylogenetic studies with available PCV-3 partial and complete sequences from around the world have revealed high nucleotide identity (>96%), although two main groups and several subclusters have been described as well. Moreover, it has been proposed the existence of a most common ancestor dated around 50 years ago. Taking into account the economic importance and the well-known effects of PCV-2 on the swine industry, a new member of the same family like PCV-3 should not be neglected. Studies on epidemiology, pathogenesis, immunity and diagnosis are guaranteed in the next few years. Therefore, the present review will update the current knowledge and future trends of research on PCV-3.

## Introduction

The evolution of emerging diseases is associated with factors embedded in the concept “host-agent-environment triangle” (1). To infect the host and cause disease, the pathogen needs to evade host defenses, which may occur through single point mutations, genome rearrangements, recombination and/or translocation (2). Genetic uniformity generated through genetic selection of the host (3) and the fact that demographic changes, intensification of farming, and international commerce have occurred markedly over the last decades, must be also considered as essential factors for the development of emerging diseases (4–6).

As well as in humans, emerging diseases drastically affect animal populations, especially food-producing animals. Livestock production in large communities (i.e., pig farms or poultry flocks) represents an excellent environment to facilitate the transmission and maintenance of huge viral populations, contributing to the pathogen evolution (through mutation, recombination and reassortment, followed by natural selection) (7–9). The intensification of livestock during the last four decades has probably been one of the main factors that contributed to the emergence of new pathogens and/or pathogen variants, leading to changes in the epidemiology and presentation of diseases (10).

The number of viral infectious diseases in swine has significantly increased in the last 30 years. Several important worldwide distributed viruses have been reported in this period, including *Porcine reproductive and respiratory syndrome virus* (PRRSV, family *Arteriviridae*), *Porcine circovirus 2* (PCV-2, family *Circoviridae*) and *Porcine epidemic diarrhea virus* (PEDV, family *Coronaviridae*). In addition to those worldwide widespread viruses, an important number of novel swine pathogens causing different types of diseases has been described (11, 12). Although their economic impact might be variable, they are considered significant infection agents and their monitoring is nowadays performed in some parts of the world. Among others, relevant examples are *Porcine deltacoronavirus* (associated with diarrhea) (12), *Senecavirus A* (causing a vesicular disease and increased pre-weaning mortality) (11), *Porcine sapelovirus* (found in cases of polioencephalomyelitis) (13), *Porcine orthoreovirus* (assumed to cause diarrhea) (14), *Atypical porcine pestivirus* (cause of congenital tremors type II) (15) and HKU2-related coronavirus of bat origin (associated with a fatal swine acute diarrhea syndrome) (16).

Besides overt emerging diseases of swine, many other novel infectious agents have been detected in both healthy and diseased animals, and their importance is under discussion. This group of agents is mainly represented by *Torque teno sus viruses, Porcine bocavirus, Porcine torovirus* and *Porcine kobuvirus*, which are thought to cause subclinical infections with no defined impact on production (13, 17, 18). An exception may be represented by *Hepatitis E virus* (HEV); although it seems fairly innocuous for pigs, it is considered an important zoonotic agent (19, 20). Recently, a novel member of the *Circoviridae* family named *Porcine circovirus 3* (PCV- 3), with unknown effects on pigs, has been discovered (21, 22).

*Porcine circovirus 3* (PCV-3) was first described in 2015 in North Carolina (USA) in a farm that experienced increased mortality and a decrease in the conception rate (21). Sows presented clinical signs compatible with porcine dermatitis and nephropathy syndrome (PDNS) and reproductive failure. In order to identify the etiological pathogen, aborted fetuses and organs from the affected sows were collected for further analyses. Whilst histological results were consistent with PCV-2-systemic disease, both immunohistochemistry (IHC) and quantitative PCR (qPCR) methods to detect PCV-2 yielded negative results. Samples were also negative for PRRSV and *Influenza A virus*. Homogenized tissues from sows with PDNS-like lesions and three fetuses were tested through metagenomic analysis, revealing the presence of an uncharacterized virus (21). Further analyses using rolling circle amplification (RCA) followed by Sanger sequencing showed a circular genome of 2,000 nucleotides. Palinski et al. (21) also performed a brief retrospective study through qPCR on serum samples from animals clinically affected by PDNS-like lesions (but negative for PCV-2 by IHC) and pigs with porcine respiratory diseases. Results revealed PCV-3 qPCR positivity in 93.75 and 12.5% of the analyzed samples, respectively (21).

Interestingly, almost concomitantly, another research group from the USA reported a clinical picture pathologically characterized by multi-systemic and cardiac inflammation of unknown etiology in three pigs of different ages ranging between 3 and 9 week-old (22). Several tissues from these animals were tested by next-generation sequencing (NGS) methods and PCV-3 genome was found. Beyond NGS, *in situ* hybridization was performed in one out of these three pigs, confirming PCV-3 mRNA in the myocardium (cytoplasm of myocardiocytes and inflammatory cells mainly, although to a very low frequency).

Based on these two initial works, the name PCV-3 was proposed as the third species of circoviruses affecting pigs, since pairwise analysis demonstrated significant divergence with the existing PCVs. The novel sequences showed < 70% of identity in the predicted whole genome and capsid protein amino acid (aa) sequence compared to the other members of the *Circovirus* genus (22). Taking into account the economic importance and the well-known effects of PCV-2 on the swine industry, a new member of the same family like PCV-3 should not be neglected. Studies on epidemiology, pathogenesis, immunity and diagnosis are guaranteed in the next few years, but the scientific community is still in its very beginning on the knowledge of this new infectious agent. Therefore, the objective of the present review is to update the current knowledge and forecast future trends on PCV-3.

## Molecular Organization of Porcine Circoviruses

*Porcine circovirus* 3 (PCV-3) belongs to the family *Circoviridae*, genus *Circovirus*. Until 2016, the *Circoviridae* family was divided into two different genera named *Circovirus* and *Gyrovirus* (23); however, on the basis of the viral structure and genome, a new taxonomical grouping has been recently established by the International Committee on Taxonomy of Virus. The genus *Gyrovirus* has been removed from the family *Circoviridae* and reassigned into the *Anelloviridae* family, and the new taxon *Cyclovirus* has been included into the *Circoviridae* family (24). This new genus is closely related with *Circovirus* genus members, with some differences in the genomic structure such as the orientation of the major open reading frames (ORFs). Moreover, viral sequences of the genus *Cyclovirus* have been reported in both vertebrates and invertebrates, including humans and other mammals (25–29), birds (30), and insects (31). Members of the *Circovirus* genus have been detected in vertebrates (32); most recently one study reported the presence of a *Circovirus* genome in invertebrates (33). One of the first *Circovirus* discovered, *Psittacine beak and feather disease virus*, was described in avian species (34) and, subsequently, several reports revealed the presence of similar virions in other species such as swine (35), fishes (36), bats (37–39), chimpanzees (40), dogs (41) humans (40), and minks (42). Since 2016, three species of porcine circoviruses have been formally accepted, including *Porcine circovirus 1* (PCV-1), PCV-2 and PCV-3 (21, 22).

Structurally, circoviruses are small single-stranded DNA (ssDNA) viruses (43), characterized by a non-enveloped virion with icosahedral symmetry, and a circular genome with a diameter ranging from 13 to 25 nm. Members of this family are constituted by 60 capsid protein subunits organized in a dodecahedral pentamer clustered unit (44). PCV-1 has a genome size ranging from 1,758 to 1,760 nucleotides (nt) (45–47), while the circular genomes of PCV-2 and PCV-3 consist of 1,766–1,769 and 1,999–2,001 nt, respectively (21, 46, 48–50).

Porcine circoviruses contain three major ORFs arranged in the strands of the replicative form (RF) (21). For PCV-1, a total of seven putative ORFs capable to encode proteins larger than 5 kDa have been predicted on both DNA strands (47), being six of them larger than 200 nt (51, 52). PCV-2 contains, besides the three major ORFs, eight more predicted ones, but just ORF4 has been characterized in more detail (53–55). PCV-3 contains so far three identified ORFs, but only ORF1 and ORF2 have been characterized. The general characteristics of the three major ORFs of PCVs are summarized in Table 1.

**Table 1:** Summary of characteristics of the three major ORFs in PCV-1, PCV-2, and PCV-3.

***Porcine circovirus***	**Size (nt)**	**ORF1**		**ORF2**		**ORF3**	
		**Protein**	**Size (aa)**	**Protein**	**Size (aa)**	**Protein**	**Size (aa)**
PCV-1	1,758–1,760	Rep Rep′	312 168	Cap	230–233	NS	206
PCV-2	1,766–1,769	Rep Rep′	314 297	Cap	233–236	NS	104
PCV-3	1,999–2,001	Rep	296–297	Cap	214	Unknown	231

*NS, Non-structural protein; nt, nucleotides; aa, amino acids*.

ORF1 encodes for Rep and Rep′ proteins involved in replication initiation, of 312 and 168 aa, respectively, in PCV-1, and of 314 and 297 aa, respectively, for PCV-2 (56). ORF1 apparently codes for a single replicase protein in PCV-3, of 296–297 aa (21, 22). ORF1 is located on the positive strand and considered the most conserved region of the circovirus genome (57). The origin of replication (*ori*), constituted by a conserved non-anucleotide motif [(T/n)A(G/t)TATTAC], is located on the same strand as ORF1 and, consequently, this frame is involved in rolling circle replication (RCR) (58).

ORF2 encodes the only structural protein (Cap). It consists of 230–233 aa for PCV-1, 233–236 aa for PCV-2 (56, 59, 60) and 214 aa for PCV-3 (21, 22). ORF2 is located on the negative DNA viral strand and Cap protein is considered the most variable (46, 61, 62), and most immunogenic (63) viral protein. Nucleotide similarity of 67% in Cap protein between PCV-1 and PCV-2 was detected through phylogenetic analyses (64); moreover, the similarity in this protein is much lower (24%) among PCV-1 and PCV-3 (22) while being 26–37% between PCV-2 and PCV-3 (21, 22).

The ORF3 is oriented in the opposite direction of ORF1, also in the negative strand, which codifies for a non-structural protein with apoptotic capacity (56, 65). The ORF3 protein consists of 206 aa for PCV-1, 104 aa for PCV-2 and 231 aa for PCV-3 (21, 66). The apoptotic activity of ORF3 protein has been described both *in vitro* and *in vivo* for PCV-1 and PCV-2 (67, 68), while its putative function in PCV-3 is still unknown.

Lastly, ORF4, also located in the negative strand, has only been described in the PCV-2 genome. This gene codifies for a protein of approximately 60 aa with anti-apoptotic function (53, 54).

Table 2 summarizes the nucleotide and amino acid raw distances (calculated by means of the median pairwise distances) among and within porcine circoviruses.

**Table 2:** Median of pairwise genetic and amino acid distance calculated for all available PCV-1, PCV-2, and PCV-3 sequences.

		**Complete genome**			**Cap**			**Rep**		
		**PCV-1**	**PCV-2**	**PCV-3**	**PCV-1**	**PCV-2**	**PCV-3**	**PCV-1**	**PCV-2**	**PCV-3**
DNA	PCV-1	0.011 [0.000–0.026]	0.228 [0.220–0.271]	0.533 [0.528–0.543]	0.017 [0.000–0.043]	0.332 [0.314–0.352]	0.598 [0.586–0.611]	0.006 [0.000–0.070]	0.174 [0.116–0.194]	0.500 [0.491–0.527]
	PCV-2	0.228 [0.220–0.271]	0.037 [0.001–0.102]	0.525 [0.518–0.544]	0.332 [0.314–0.352]	0.057 [0.000–0.172]	0.547 [0.539–0.569]	0.174 [0.116–0.194]	0.022 [0.000–0.056]	0.495 [0.485–0.520]
	PCV-3	0.533 [0.528–0.543]	0.525 [0.518–0.544]	0.009 [0.000–0.024]	0.598 [0.586–0.611]	0.547 [0.539–0.569]	0.014 [0.000–0.028]	0.500 [0.491–0.527]	0.495 [0.485–0.520]	0.006 [0.000–0.034]
Amino acid	PCV-1	NA	NA	NA	0.028 [0.000–0.071]	0.303 [0.283–0.346]	0.748 [0.732–0.760]	0.006 [0.000–0.075]	0.147 [0.088–0.194]	0.583 [0.577–0.607]
	PCV-2	NA	NA	NA	0.303 [0.283–0.346]	0.055 [0.000–0.177]	0.689 [0.681–0.736]	0.147 [0.088–0.194]	0.009 [0.000–0.075]	0.574 [0.564–0.602]
	PCV-3	NA	NA	NA	0.748 [0.732–0.760]	0.689 [0.681–0.736]	0.012 [0.000–0.035]	0.583 [0.577–0.607]	0.574 [0.564–0.602]	0.003 [0.000–0.031]

*The distance range is reported between brackets after removal of the lower and upper 0.1 percentile. This measure was selected to exclude extreme values, which could be due to poor quality of some sequences challenging to be detected during alignment inspection. NA, non-applicable*.

**Figure 1:**
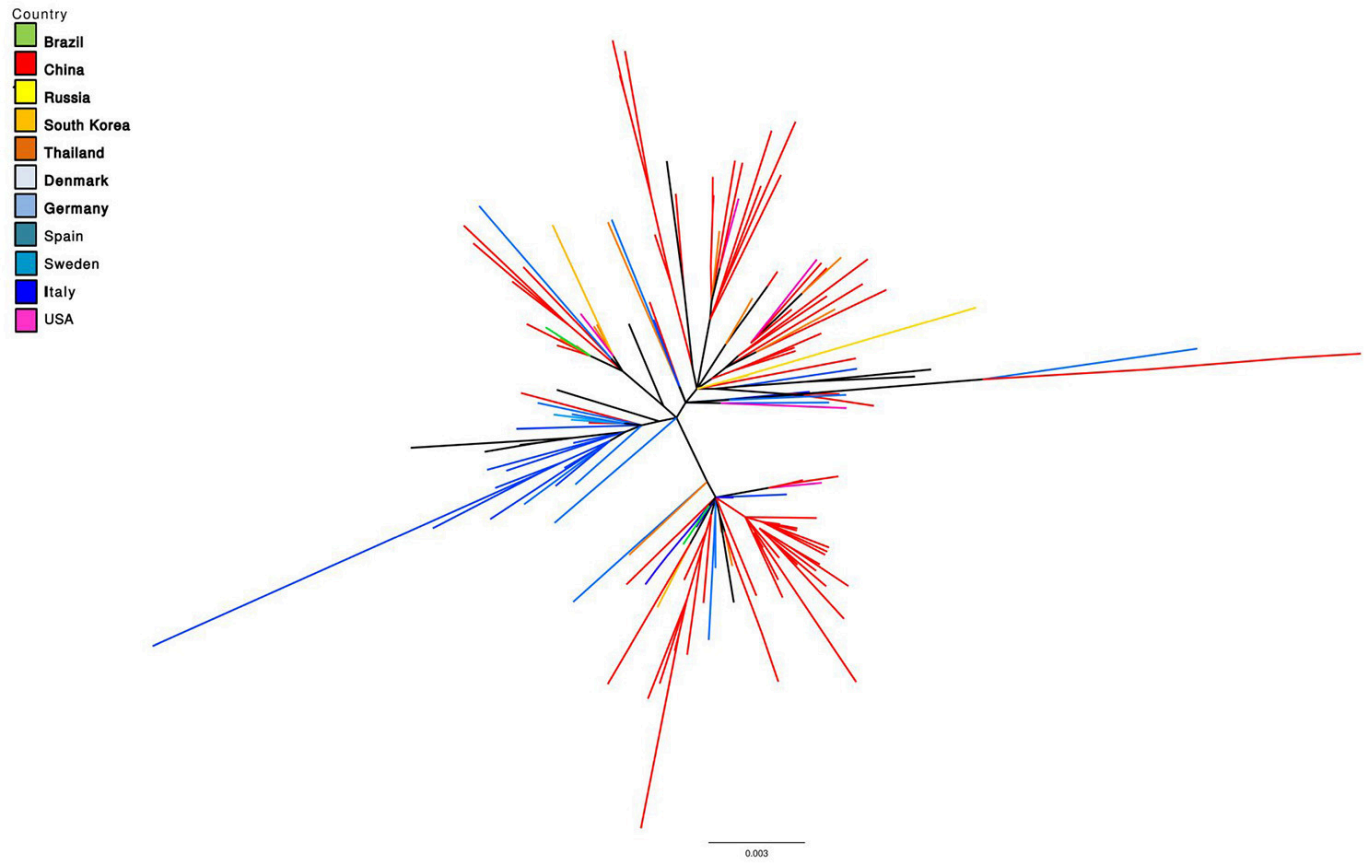
Maximum Likelihood unrooted phylogenetic tree reconstructed based on all available PCV-3 complete genome sequences (retrieved on October 2018). Tree terminal branches have been color coded according to the corresponding collection country. Black color in terminal branches indicates other countries not included in the list.

The similarity between PCV-3 sequences ranges from 97 to 100% throughout the analyzed years and tested countries (48, 69–71). Phylogenetic analyses suggested two main groups classified as PCV-3a and PCV-3b and several sub-clusters (48, 72, 73), based on differences found between both groups in the aa sites 122 and 320 (S122A and A320V). In fact, certain antigenicity differences among groups have been proposed (74), although it is still too early to discuss about potential different genotypes or subgroups for PCV-3. Additionally, the progressive increase in sequence availability is revealing the presence of other branching patterns, which hardly fit with the “two genotype” classification. Therefore, similarly to PCV-2, a higher heterogeneity might be found in the future. A phylogenetic tree including full-length sequences of PCV-3 is depicted in Figure 1.

## Epidemiology

**Figure 2:**
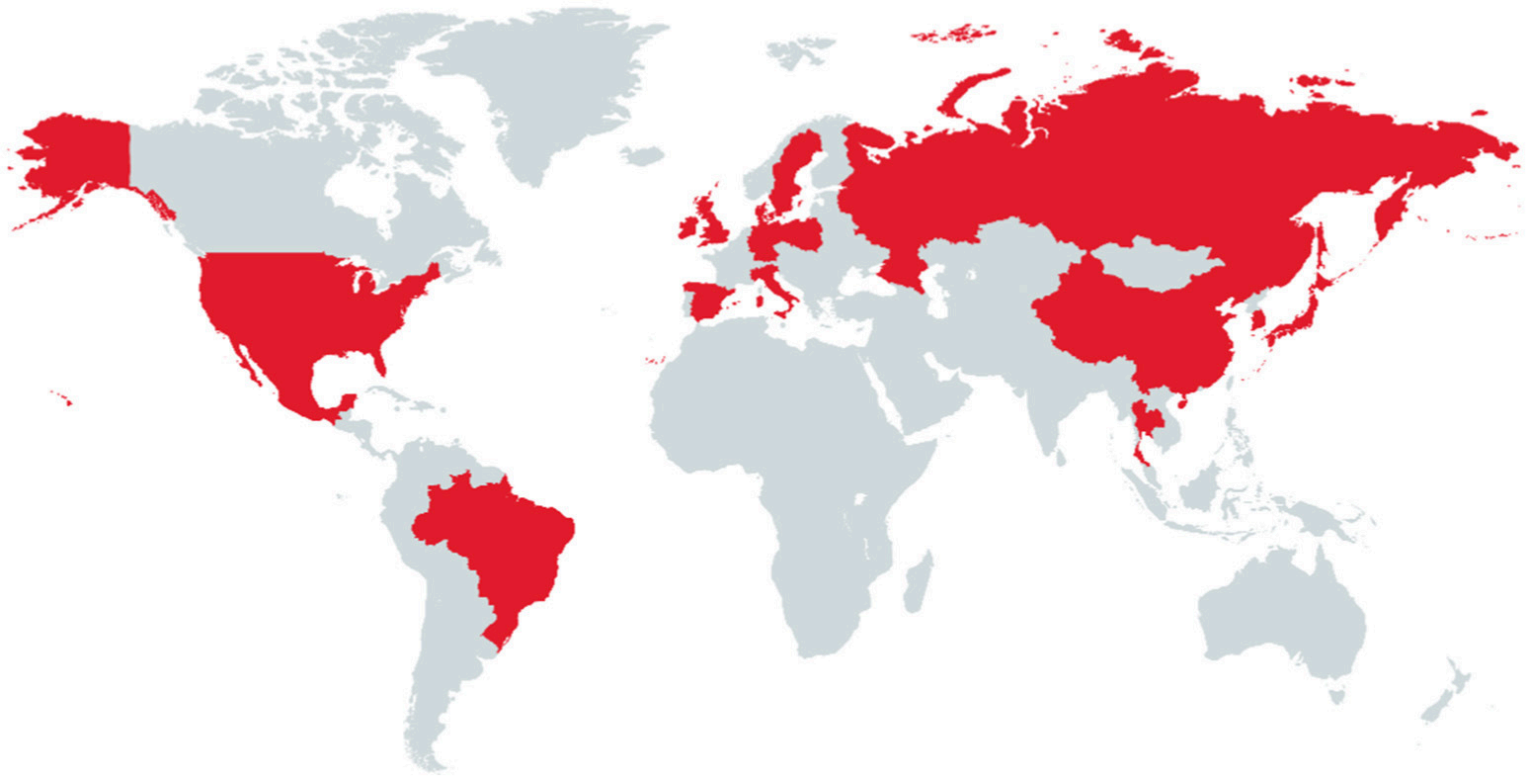
Countries in red are those that have been so far reported PCV-3 PCR positive samples in domestic pig.

After the first description reported from the USA, several countries located in Asia, Europe and South America (Figure 2) have demonstrated the presence of PCV-3 genome in domestic pig (70, 73, 75–80).

PCV-3 genome has been detected at all tested ages, including sows, mummified fetuses and stillborn (21, 79, 81). The frequency of viral detection found by PCR in pigs is variable according to the collected samples around the world (Table 3). A lower frequency of PCV-3 PCR positivity has been detected in lactating pigs when compared with nursery and fattening ones; the highest prevalence was found in animals after weaning (48, 77, 82). However, these studies included different pigs from fairly limited age-groups and not the same animals over time. In a very recent work performed on longitudinally sampled pigs in Spain (83), PCV-3 DNA was found at all age-groups in four tested farms, and the frequency of infection was not clearly dominant at any age. Also, PCV-3 has been detected at moderate to high rate in sera pools from sows in Poland (77) and Thailand (84).

**Table 3:** Reports describing PCV-3 frequency of detection on different countries and sample types.

**References**	**Country**	**Sample type**	**PCV-3 positive (*n*)**	**Tested samples (*n*)**	**Frequency of detection (%)**
Collins et al. (85)	Ireland	Tissue and feces	52	313	16.61
Fu et al. (73)	China	Tissue and stillborn	76	285	26.67
Kwon et al. (82)	South Korea	Oral fluid	159	360	44.17
Ku et al. (70)	China	Tissue, stillborn, semen and serum	77	222	34.68
Palinski et al. (21)	USA	Serum	47	150	31.33
Stadejek et al. (77)	Poland	Serum	55	215	25.58
Xu et al. (86)	China	Tissue and serum	53	170	31.18
Zhai et al. (87)	China	Tissue and serum	84	506	16.60
Zheng et al. (71)	China	Tissue	132	222	59.46
Wen et al. (88)	China	Tissue and serum	50	155	32.26
Klaumann et al. (81)	Spain	Serum	75	654	11.47
Franzo et al. (89)	Italy	Sponge sample	2	4	50.00
Franzo et al. (76)	Denmark	Tissue and serum	44	78	56.41
Franzo et al. (76)	Italy	Tissue and serum	36	91	39.56
Franzo et al. (76)	Spain	Serum (pools)	14	94	14.89
Hayashi et al. (90)	Japan	Tissue	7	73	9.59
Kedkovid et al. (84)	Thailand	Colostrum	17	38	44.74
Kedkovid et al. (91)	Thailand	Tissues and serum	33	103	32.04
Sun et al. (78)	China	Tissue	13	200	6.50
Zou et al. (69)	China	Serum	62	190	32.63
Zhao et al. (92)	China	Tissue	40	272	14.71
Ye et al. (93)	Sweden	Tissue	10	49	20.41
Kim et al. (94)	Korea	Serum	37	286	17.9
Kim et al. (94)	Korea	Tissue	20	296	6.8

PCV-3 genome has been detected by PCR in oral fluids and nasal swabs (76, 82) as well as in feces (85, 95), semen (70), and colostrum (84). Kedkovid et al. (84) found a positive correlation between detection in serum samples and in colostrum, suggesting that the colostrum is influenced by the viremic stage of the sow. No specific studies have been performed on the virus detection in the environment, but one study indicates that the virus was found in 2 out of 4 sponges used for sampling pig transporting trucks after sanitation (89).

Besides domestic pigs, PCV-3 infects wild boar. Viral DNA sequences retrieved from wild boar showed more than 98% similarity with the available sequences from domestic pigs (95, 96). The prevalence found in tested serum samples was similar or higher than that found in domestic pigs, ranging from 33 to 42.66%. Additionally, infection susceptibility was associated with the age in both studies; juvenile animals were statistically less often PCV-3 PCR positive than the older ones. In fact, a potential reservoir role of the wild boar with respect to PCV-3 infection has been suggested (95, 96).

PCV-3 seems to be restricted to *Suidae* species. However, PCV-3 genome has been found in 4 out of 44 (9.09%) serum samples of dogs from China. The authors suggested that the virus might infect, therefore, non-porcine species (97). To date, there is no further evidence regarding susceptibility to PCV-3 infection in other species.

## Disease Association With PCV-3

PCV-3 has been detected in pigs with different clinical/ pathological conditions, such as respiratory, reproductive, gastrointestinal and neurological disorders; however, the virus has been also detected in apparently healthy animals (21, 71, 98). The conditions in which PCV-3 has been found are summarized in Table 4. Noteworthy, in most of these scenarios there are not complete diagnostic studies, but only the detection of the viral genome in a number of pigs affected by different clinical signs. Even though the viral genome was detected, it is worthy to state that it does not imply a causative role of PCV-3 in the observed condition. Thus, this section compiles the peer-reviewed papers, reporting PCV-3 DNA detection in different disease scenarios.

**Table 4:** Clinical signs reported in PCV-3 PCR positive animals according to production phase in different clinical/pathological scenarios.

**Disorders**	**Production phase**	**Clinical signs - disease**	**Control group-healthy animals**	**Reference**
Reproductive	Sows	•Increase in the sow mortality; decrease in the conception rates; mummified fetuses •Aborted fetuses, stillborn •Abortion, mummified fetuses; reproductive failure; decrease of neonatal rate	NA NA NA	Palinski et al. (21) Faccini et al. (75) Ku et al. (70)
Respiratory	Lactation Weaning Weaning Fattening Fattening	•Dyspnea •Anorexia, fever, icterus, abdominal breathing •Cough, softly panting, abdominal breathing •Respiratory signs •Porcine respiratory disease complex (PRDC)	NA NA Yes* NA NA	Phan et al. (22) Shen et al. (99) Zhai et al. (87) Phan et al. (22) Kedkovid et al. (91)
Cardiovascular	Weaning	•Anorexia, weight loss, swollen joints	NA	Phan et al. (22)
Gastrointestinal	Weaning	•Diarrhea	Yes*	Zhai et al. (87)
Systemic	Weaning	•Wasting •Periweaning failure-to-thrive syndrome (PFTS)	Yes* Yes*	Stadejek et al. (77) Franzo et al. (100)
Neurological	Lactation Lactation	•Neurological signs •Congenital tremors	NA NA	Phan et al. (22) Chen et al. (101)
Others	Fattening Sows	•Rectal prolapse •PDNS	NA NA	Phan et al. (22) Palinski et al. (21)

NA, not available in the published study;

* *PCV-3 positivity in lower frequency than diseased animals*.

The amount of viral DNA in serum samples (10^2^-10^7^ copies/mL) (21) and tissues (10^4^-10^11^ copies/mg) (86, 91) in postweaning pigs and adults was rather variable, as well as in stillborn or fetal tissues (10^6^-10^9^ copies/mg) (21, 75). In most of these cases, the number of PCV-3 genome copies should be considered moderate to low (21, 91). In addition, detection was possible in some instances, but the viral load was below the limit of quantification of the qPCR, which may emphasize the subclinical nature of the infection in these cases (48, 81). An association between high viral load and severity has been demonstrated for other porcine circovirus (PCV-2), especifically under PCV-2-SD (102) and PCV-2-reproductive disease (103) scenarios. However, the meaning of a given genome viral load for PCV-3 in healthy or diseased pigs is still to be elucidated.

### Reproductive Disease

PCV-3 genome was initially retrieved from sows with clinical signs compatible with PDNS in USA. In the affected farm, a decrease of 0.6% in the conception rate was found while the sow mortality showed a 10.2% increase (21). In China, PCV-3 was found in serum samples from sows with reproductive problems characterized by acute loss of neonatal piglets (70). Moreover, a comparative study between healthy sows and sows with a clinical picture characterized by chronic reproductive failure (including increase in abortion and sow mortality rates) revealed that PCV-3 positivity was higher in affected sows (39 out of 84, 46.42%) than in healthy ones (23 out of 105, 21.9%) (69). Viral genome has also been found in tissues from stillborn in farms experiencing reproductive failure in China (69–71) and Korea (94).

### Respiratory Disease

PCV-3 DNA was also detected in pigs with respiratory disorders, as already indicated in the first report of this virus (21). Two more studies reported PCV-3 genome in animals from China with abdominal breathing and lesions including lung swelling and congestion (87, 99). More recently, the viral genome has been detected in fattening pigs from Thailand suffering from porcine respiratory disease complex (PRDC), characterized by coughing, dyspnea, fever and anorexia; the prevalence was higher in diseased animals (60%; 15 out of 25) than in healthy ones (28%; 7 out of 25) (91).

### Other Conditions

Multisystemic inflammation and myocarditis were initially linked with the presence of PCV-3 (22). One single study described PCV-3 in weaned pigs that suffered from gastro-intestinal disorders (diarrhea), showing higher prevalence in pigs with clinical signs (17.14%, 6 out of 35) compared to those with non-diarrhea signs (2.86%; 1 out of 35) (87). In another report, animals with congenital tremors were analyzed and PCV-3 was the only pathogen found in the brain, with high amount of viral DNA (101).

### Healthy Animals

A number of studies found PCV-3 in apparently healthy animals (69, 76, 81, 87, 93), which makes much more complicated the overall interpretation of this virus as potential causative agent of disease.

### Co-infections

Whilst the initially PCV-3 PCR positive cases were negative for three of the most important swine infectious agents (PCV-2, PRRSV, and *Porcine parvovirus*, PPV) (21, 22, 87), subsequent studies revealed frequent co-infection with other viruses. All pathogens found in co-infections with PCV-3 are summarized in Table 5.

**Table 5:** Pathogens present in PCV-3 PCR positive samples/cases.

**Pathogen**	**Frequency of co-infection (percentage)**	**Reference**
PCV-2	38/200 (19%) 28/40 (70%) 35/222 (15.77%) 13/46 (28.26%) 1/8 (12.5%)	Sun et al. (78) Zhao et al. (92) Ku et al. (70) Kim et al. (104) Kedkovid et al. (91)
	11/57 (19.3%)	Kim et al. (94)
PRRSV	1/8 (12.5%)	Kedkovid et al. (91)
	25/57 (43.86%)	Kim et al. (94)
*Torque teno sus virus* (TTSuV1 and 2)	66/132 (50%)	Zheng et al. (78)
*Classical swine fever virus* (CSFV)	108/200 (54%)	Sun et al. (78)
*Porcine bocavirus* (PBoV)	NA	Chen et al. (101)
*Porcine epidemic diarrhea virus* (PEDV)	NA	Chen et al. (101)
*Atypical porcine pestivirus* (APPV)	NA	Chen et al. (101)
*Porcine deltacoronavirus* (PDCoV)	NA	Chen et al. (101)
*Porcine kobuvirus* (PKV)	NA	Chen et al. (101)
*Porcine pseudorabies virus* (PRV)	NA	Chen et al. (101)
*Porcine sapelovirus* (PSV)	NA	Chen et al. (101)
*Porcine parvovirus* (PPV)	NA	Franzo et al. (100)
*Ungulate bocaparvovirus 2* (BoPV2)	NA	Franzo et al. (100)
*Pasteurella multocida*	NA	Kedkovid et al. (91)
*Haemophilus parasuis*	NA	Phan et al. (22)
*Streptococcus suis*	NA	Phan et al. (22)
*Mycoplasma hyorhinis*	NA	Phan et al. (22)

*NA, not available in the published study*.

It is still too early to establish the overall picture of PCV-3 infection, since it is a widespread virus in healthy animals. Therefore, the likelihood of disease may not depend on its presence only, but other factors may serve as illness triggering factors or up-regulate its replication under disease scenarios.

## Laboratory Tools to Detect PCV-3

The detection of the virus is currently based on molecular techniques such as conventional PCR and qPCR and its characterization by Sanger sequencing or NGS. In fact, the first PCV-3 complete genome was identified by NGS, and subsequently Sanger sequencing has been systematically applied to obtain novel PCV-3 sequences. Several primer pairs and probes have been designed for these molecular techniques (21, 89, 101). Moreover, a duplex qPCR for the simultaneous detection of PCV-2 and PCV-3 has been also attempted (105).

*In situ* hybridization, a technique used to detect viral genome on histological tissue sections, has been performed in two studies (22, 91). However, the technique is not yet completely standardized, since it is still used in minimal number of laboratories worldwide and a thorough description of the infected cell types is still missing.

A minimum number of studies showed the development and validation of serological tests. Two reports have published limited information about indirect enzyme-linked immunosorbent (ELISA) tests using recombinant PCV-3 Cap protein (21, 106). More recently, a PCV-3 specific monoclonal antibody has been produced, presumably working on formalin-fixed, paraffin-embedded tissues by means of immunohistochemistry (72).

Infection of cell cultures with PCV-3 tissue homogenates has been attempted in PK-15 (21, 75) and swine testicle cells (ST) (21) without success. The cells were observed for cytopathic effects and monitored by qPCR for viral growth. However, the Ct-values did not increase at each cellular passage and no cytopathic effect was observed (21, 75). Therefore, there is not any PCV-3 isolate so far available.

Definitely, in order to elucidate the PCV-3 pathogenesis, further establishment of laboratory techniques such as viral isolation, serology, and detection of viral components in tissues is needed. In consequence, the potential association of PCV-3 with any clinical condition, if any, is difficult to be demonstrated due to existing technical limitations.

## Knowledge Gaps of PCV-3 Infection

### PCV-3 As a Cause of Disease

Porcine circoviruses (PCVs) are ssDNA ubiquitous viruses, widespread worldwide in the domestic pig population (107). Two species were known to infect *Suidae* species before 2015: PCV-1, considered non-pathogenic, and PCV-2, the cause of one of the most devastating porcine diseases, PCV-2-SD. PCV-3 represents an expansion of the swine virosphere within the *Circoviridae* family, but the up-to-date knowledge is still very limited and there is not yet any clue on its potential pathogenesis or disease causation role. It is at least curious that 20 years ago there were serious doubts about PCV-2 as a cause of an overt disease characterized by severe lesions and high mortality (108), while nowadays PCV-3 has been found within a number of clinical conditions and putative association has been established from the very beginning (21, 22).

Current literature has already reported the presence of PCV-3 in animals affected by different clinical pictures, although just few of them included healthy control groups (71, 76, 87, 91). In all studies, the frequency of PCV-3 detection in diseased animals was higher; although these results did not prove any disease causality, at least open the avenue to definitively ascertain its role in clinical/pathological manifestations. Further studies on potential disease association of PCV-3 are needed.

### Pathogenesis

No data is available regarding the pathogenesis of PCV-3 infection. The lack of virus isolation has impeded the establishment of an infection model to date. It is known that PCV-3 can be found in different tissues of domestic pig and wild boar (86, 87, 95), indicating the systemic nature of the infection. However, the point of viral entry, primary replication, organic distribution and persistence are still unsolved issues. PCV-3 has been found in feces, nasal swabs, oral fluids, and trucks transporting pigs (82, 85, 95), which allows speculating that horizontal transmission through direct contact is probably an important route. Detection of viral genome in fetuses and stillborn from farms with history of reproductive failure (21, 70, 75), as well as in semen and colostrum, points out also to vertical transmission as another likely route. Definitively, more studies are needed to ascertain the potential excretion routes of this virus.

### Co-infections

Co-infection of PCV-3 with both PCV-2 and PRRSV has been reported (70, 78, 91, 92, 94). In fact, this was expected since both well-known pathogens are widespread in the pig population (109–111). Noteworthy, it is known that both PCV-2 and PRRSV are able to affect the immune system and, therefore, co-infections with these viruses are not unusual (112, 113). Other pathogens were also detected in PCV-3 PCR positive samples (78, 114). Very recently, PCV-3 has been found by NGS approach in pigs affected by periweaning failure-to-thrive syndrome in co-infection with PPV and *Ungulate bocaparvovirus 2* (100). Since experimental and field studies demonstrated that co-infection with PPV increase the effect of PCV-2 in causing PCV-2-SD (115), at this point it cannot be ruled out that a similar effect may occur with PCV-3. Further investigations are needed to determine whether PCV-3 might act as a secondary agent up-regulating its replication once pigs are immunosuppressed or immunomodulated, or whether the frequency of co-infection is independent of the immune system affection.

### Age of Infection and Transmission

Although PCV-3 genome has been detected at higher prevalence in weaned pigs (48, 77, 82), only one study has monitored PCV-3 infection longitudinally (83). In this study, PCV-3 was found in pigs at all ages with a similar frequency. This infection dynamics contrasts with that of PCV-2, which infects pigs mainly between five and 12 weeks of age, and rarely in animals at the lactation phase (116–118). This is explained by the fact that colostrum antibodies are protective against infection and then decline during the lactation and weaning phases. Once maternally derived antibodies waned, an infection is followed by active seroconversion (117–119). This seroconversion usually occurs between 9 and 15 weeks of age and the antibodies may last until 28 weeks of age at least (117, 120–122). Regrettably, information about infection in sows, maternally derived immunity and how protective the immunity might be against PCV-3 is completely lacking at this moment. It is known that PCV-3 can be found in colostrum (84), implying the possibility of vertical transmission (sow to piglet) and emphasizing the potential importance of early infections. Again, available information regarding these issues on PCV-3 is still to be generated.

### Persistent or Long Lasting Infection

One study performed in samples from captured and re-captured wild boar revealed long-lasting infection (potential persistent infection), since the virus was detected during a period of at least 5–7 months in few animals (95). Susceptibility of wild boar to PCV-3 was not a surprise, since this species shows susceptibility to several pathogens that affect humans and animals (123), including PCV-2; moreover, the wild boar can also develop PCV-2-SD (124). Taking into account the potential long period of infection observed in some animals and even a higher overall prevalence in wild boar when compared with domestic pigs, such potential reservoir role deserves further investigations (95, 96).

### Spectrum of Species Infected and Public Health Issues

Infection of PCV-3 in other non-*Suidae* species is, at this point, still to be demonstrated. Although PCV-3 DNA has been found in sera from dogs in China (97), the lack of other detection techniques able to confirm a true infection with this virus prevents the assumption of multiple species susceptibility.

Another interesting aspect yet currently unknown is the potential impact of PCV-3 on public health. DNA from PCV-1 and PCV-2 has been found in vaccines intended for use in humans (125), probably associated to the use of reagents from swine origin in the vaccine manufacturing. At this point, no information regarding PCV-3 and its role as a contaminant of human medicines do exist. On the other hand, porcine circoviruses belong to a group of microorganisms that still has not been fully addressed in terms of risk evaluation for xenotransplantation (126), so, PCV-3 should be also *a priori* added to such list.

### Origin, Evolution, and Phylogeny

Palinski et al. (21) conducted a brief study in paraffin fixed tissues from 2010 to 2016 in North America and results showed a high percentage of PCR positivity in these samples, suggesting that the virus emerged before the year of its discovery. In fact, PCV-3 has been already demonstrated retrospectively in Sweden in 1993 (93) and Spain (81) and China in 1996 (78), indicating that this is not a new virus and it has been circulating during several decades in domestic pigs. Moreover, PCV-3 has been detected in the oldest samples so far tested in these studies, suggesting that this virus could have been infecting pigs for even a longer period. However, these findings cannot be assumed as a proof of non-pathogenicity, especially when mirroring another closely-related circovirus, PCV-2. Although this latter virus was initially detected in association with disease by mid-late 1990s, retrospective studies showed evidence of pig infection a number of decades before (120, 127–129). In fact, in most of these investigations, evidence of PCV-2 infection coincided with the very first investigated year, suggesting again that PCV-2 might be even an older circulating virus. In addition, a retrospective study on PCV-3 conducted in samples of wild boar from Spain during a 14-year period (95) detected the virus in the first tested year (2004). Overall, obtained data confirmed that PCV-3 is not a new virus and has been circulating for a fairly, non-determined long time in swine and wild boar populations. In fact, the most common ancestor of PCV-3 was estimated to be originated approximately in 1966 (73, 130).

Genetic characterization of PCV-3 is mainly done through Sanger sequencing. Phylogenetic analyses of PCV-3 genomes available from the GenBank indicate they are part of different clusters. However, nucleotide identity among these sequences is really high (>97%). In consequence, it seems that PCV-3 has remained fairly stable over the years without an independent molecular evolution according to specific areas of the world. Moreover, these findings do not point out a high mutation rate as has been suggested (48, 131). If such mutation rate were high, it would have generated a higher genomic heterogeneity, which should have been detected at least in the performed retrospective studies accounting for more than 20 years. Further studies on the evolution on PCV-3 are crucial to solve out these controversies.

The first metagenomics sequence available from PCV-3 revealed low identity with *cap* and *rep* genes of PCV-1 and PCV-2 and a closer identity with other Circoviruses such as *Canine circovirus* (21, 22) and *Barbel circovirus* (71). The *Circovirus* genus members are able to infect a wide range of hosts, and cross-species transmission has also been reported (40). Franzo and collaborators (132) hypothesized the possibility of PCV-3 being the product of recombination related with a host jump. The analysis of genome composition of PCV-3 found the *rep* gene closely related with that of bat circoviruses and *cap* gene with that of avian ones (132). Recently, novel circoviruses isolated in civets, showing higher similarity in terms of aa sequence in Rep protein with PCV-3, have been described (133). The increasing new data should be useful to clarify the relationships and origin of this virus. On the other hand Fux et al. (48) found nucleotide changes, which resulted in two aa alterations in ORF1/ORF2 and ORF3 (A24V and R27K), between the two proposed genotypes (PCV-3a and PCV3b). Li et al. (131) also suggested two groups with two individual subclades termed PCV-3a-1 and PCV-3a-2. The aa site 24 from ORF2, predicted to be under positive selection, was suggested to be located in a potential epitope region. The presence of possible genotypes was also suggested in other studies (73, 76). However, considering the high similarity found in partial or complete PCV-3 sequences (>98% in most of the cases), the importance of determining genotypes or groupings at this stage seems poorly relevant. Due to the sensitivity limitations of Sanger sequencing, it must be emphasized the need to apply NGS technology to discover minor variants, which might unravel the presence of quasispecies undetected by the currently used technology.

## Conclusions

*Porcine circovirus 3* is a recently discovered virus widespread in both domestic pigs and wild boar population. The virus can be found at all tested ages and few animals may display a persistent infection. Although the virus has been found in several clinical and pathological conditions, a definitive proof of its pathogenicity is still lacking. Phylogenetic information available to date indicates a low genetic variability of PCV-3 in comparison with other single stranded-DNA viruses and indicates that the virus genome has been relatively stable across the years.

## Author Contributions

FK and JS did the majority of the writing and communicated with the coauthors to coordinate the document editing. JS designed the outline of the manuscript. GF provided the phylogenetic analyses. FC-F, GF, MS, and JN revised the manuscript, did partial writing and approved the final version for publication.

### Conflict of interest statement

The authors declare that the research was conducted in the absence of any commercial or financial relationships that could be construed as a potential conflict of interest.
